# Improving the yeast two-hybrid system with permutated fusions proteins: the Varicella Zoster Virus interactome

**DOI:** 10.1186/1477-5956-8-8

**Published:** 2010-02-15

**Authors:** Thorsten Stellberger, Roman Häuser, Armin Baiker, Venkata R Pothineni, Jürgen Haas, Peter Uetz

**Affiliations:** 1 Institute of Toxicology and Genetics, Karlsruhe Institute of Technology, PO Box 3640, D-76021 Karlsruhe, Germany; 2 Max-von-Pettenkofer Institute, Ludwig-Maximilians-University of Munich, Pettenkoferstrasse 9a, 80336 München, Germany; 3 Division of Pathway Medicine, University of Edinburgh, 49 Little France, Crescent, Edinburgh EH16 4SB, UK; 4 J Craig Venter Institute (JCVI), 9704 Medical Center Drive, Rockville, MD 20850, USA

## Abstract

**Background:**

Yeast two-hybrid (Y2H) screens have been among the most powerful methods to detect and analyze protein-protein interactions. However, they suffer from a significant degree of false negatives, i.e. true interactions that are not detected, and to a certain degree from false positives, i.e. interactions that appear to take place only in the context of the Y2H assay. While the fraction of false positives remains difficult to estimate, the fraction of false negatives in typical Y2H screens is on the order of 70-90%. Here we present novel Y2H vectors that significantly decrease the number of false negatives and help to mitigate the false positive problem.

**Results:**

We have constructed two new vectors (pGBKCg and pGADCg) that allow us to make both C-terminal fusion proteins of DNA-binding and activation domains. Both vectors can be combined with existing vectors for N-terminal fusions and thus allow four different bait-prey combinations: NN, CC, NC, and CN. We have tested all ~4,900 pairwise combinations of the 70 Varicella-Zoster-Virus (VZV) proteins for interactions, using all possible combinations. About ~20,000 individual Y2H tests resulted in 182 NN, 89 NC, 149 CN, and 144 CC interactions. Overlap between screens ranged from 17% (NC-CN) to 43% (CN-CC). Performing four screens (i.e. permutations) instead of one resulted in about twice as many interactions and thus much fewer false negatives. In addition, interactions that are found in multiple combinations confirm each other and thus provide a quality score. This study is the first systematic analysis of such N- and C-terminal Y2H vectors.

**Conclusions:**

Permutations of C- and N-terminal Y2H vectors dramatically increase the coverage of interactome studies and thus significantly reduce the number of false negatives. We suggest that future interaction screens should use such vector combinations on a routine basis, not the least because they provide a built-in quality score for Y2H interactions that can provide a measure of reproducibility without additional assays.

## Background

The yeast two-hybrid (Y2H) system has been among the most powerful methods to identify protein-protein interactions. However, it has also been criticized for generating large numbers of false positive and false negative data. While false positives can be minimized by including controls, retesting by independent methods, and bioinformatic filtering, false negatives pose a much bigger problem. In fact, many interactions must go undetected in two-hybrid screens because of the sterical constraints the system involves: the two fusion proteins must interact with each other, their interaction interfaces must be exposed, and all components must be oriented on the DNA in a way that the activation domain of the prey can productively interact with the transcriptional machinery.

On top of sterical constraints, small variations of the Y2H system can lead to large differences in resulting interactions [1]. As no other large-scale screen has used multiple Y2H systems, not much attention has been paid to this phenomenon. Here we investigate such multi-system screens to characterize the interactome of Varicella-Zoster Virus (VZV). While many smaller viruses and their interactions have been studied, larger viruses have been analyzed only recently [2,3]. The five herpesviruses studied by Fossum et al. (i.e. HSV1, EBV, mCMV, VZV, KSHV) have been the only herpesviruses studied systematically for pairwise intraviral interactions [4]. However, we also know that the few studies that have been done resulted in markedly different interactions. For example, two systematic screens of protein-protein interactions among proteins of Kaposi-Sarcoma Herpesvirus (KSHV) yielded very little overlap [5,6]. Clearly, slight variations of vectors, strains, or assay conditions can strongly affect the resulting interactions, even when identical proteins are used.

**Figure 1:**
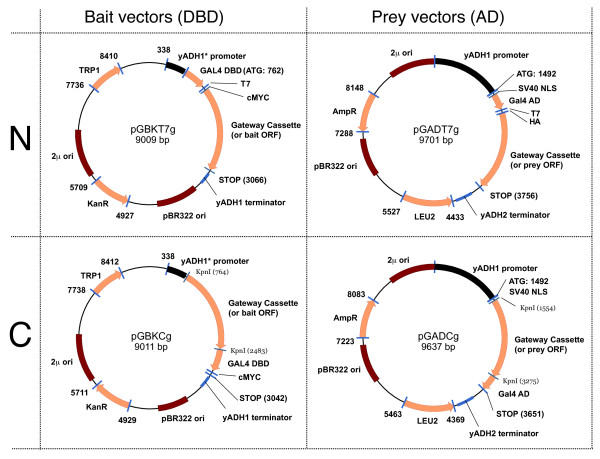
**Vectors described in this study including their parental vectors**. pGBKT7g and pGADT7g generate N-terminal fusions of DNA-binding (DBD) and activation domain (AD) fusions, respectively. The new vectors pGBKCg and pGADCg fuse DBD and AD at the C-terminus of inserted ORFs. Note that both pGBK vectors use a truncated version of the ADH promoter (indicated by *) which may reduce expression levels and thus interaction signals [26,27].

**Figure 2:**
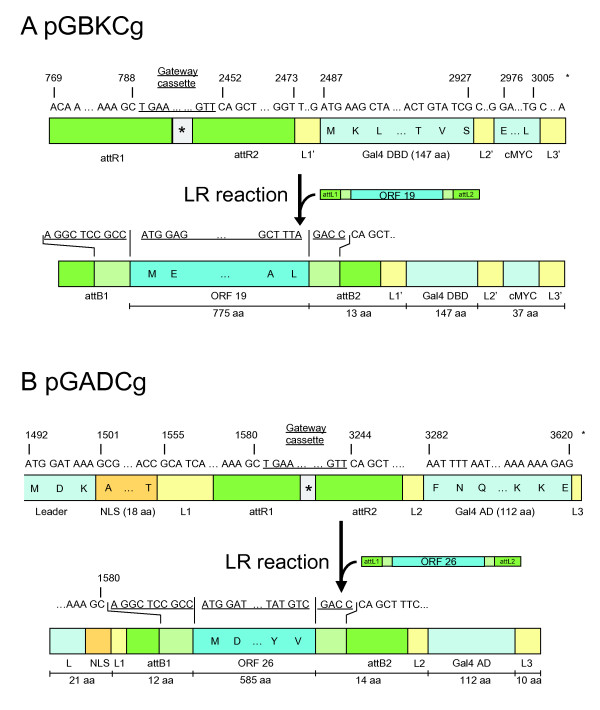
**Cloning sites and fusion products**. (A) pGBKCg generates a C-terminal fusion of the Gal4 DBD separated by a 13 amino acid linker from the N-terminally fused ORF (here: VZV ORF 19). A MYC tag is embedded in a C-terminal 37 amino acid tail. (B) pGADCg generates a C-terminal fusion of the Gal4 AD. The cloned ORF (here: VZV ORF 26) is preceded by a 33 amino acid sequence that contains the nuclear localization signal (NLS). The NLS of pGBKC is part of the DBD. A 14 amino acid linker separates the ORF from the Gal4 AD. The HA tag of pGADCg has been shortened to seven amino acids so that it may not be recognized by anti-HA antibodies. During the LR reaction the Gateway cassette in both vectors will be replaced by the sequence of interest plus additional nucleotides from the entry clone (i.e. the underlined sequence will be replaced). For further technical details see the Gateway manuals at http://www.invitrogen.com. pGBKCg and pGADCg have been deposited with and are available from Addgene http://www.addgene.com. Their sequences have been deposited with GenBank under accession numbers FJ696409 (pGBKCg) and FJ696408 (pGADCg). pGBKT7g and pGADT7g are available from the authors.

Here we present new vectors that vary one parameter of Y2H screening, namely the location of the Gal4 DNA-binding (DBD) and activation domain (AD) (Figures 1 and 2). Traditionally, Y2H vectors carry their cloning sites C-terminal of their DBD and AD so that the bait and prey proteins are fused C-terminally to these domains. Several vectors have been described that use C-terminal fusions of AD and DBDs (e.g. [7-10]). These studies have shown that the location of the fusion site is critical when selected protein pairs were tested (e.g. [7]). However, previously made vectors are not easily compatible with high-throughput cloning because they require conventional restriction digests and cloning. As a consequence, it remained unclear how N- or C-terminal fusions compare when larger sets of proteins or whole genomes are analyzed in standardized Y2H assays.

## Results

### Interactions resulting from different vectors

**Figure 3:**
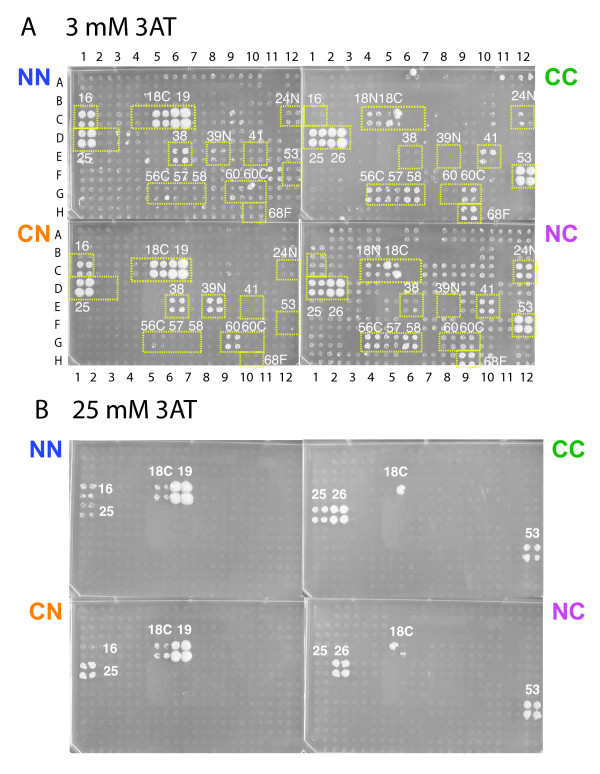
**N- and C-terminal vectors detect different interactions**. Y2H screens of the four different vector combinations showing the differences on 3 mM (A) and 25 mM 3AT (B). The same bait, ORF19 (Uniprot accession P09248) was used as bait with N-terminally and C-terminally fused DNA-binding and activation domains and screened against a whole-genome array of Varicella Zoster Virus (VZV). The N-terminal bait and prey constructs (in pGBKT7g, pGADT7g, NN) show markedly different interaction patterns compared to the C-terminal constructs cloned into pGBKCg and pGADCg (CC). NC and CN combinations show yet different interactions. Preys are indicated by their ORF number, e.g. the bait ORF19 is the large ribonucleotide reductase (RNR) subunit which is known to interact with itself and the small RNR subunit (ORF18 = Uniprot P09247). Note that N and C labels near yeast colonies indicate N- and C-terminal protein fragments, not AD or DBD fusions (e.g. 18C and 18N are N- and C-terminal domains of ORF18). A complete list of interactions is provided in Additional file 1: Table S1. The sequences of all proteins are listed in Additional file 1: Table 4.

Each of the four bait/prey vector combinations produced significantly different interactions. For example, the screens with bait ORF19, the large subunit of ribonucleotide reductase, produced a total of 17 interactions (of 15 distinct proteins), of which only two were found in all four combinations (namely ORF25 and ORF18C) (Figure 3A). Five interactions were found with the N-terminal fusions (in pGBKT7g and pGADT7g) while 11 (3 strong + 8 weak) interactions resulted from the screens with the C-terminal fusions (in pGBKCg and pGADCg). The CN and NC combinations generated 7 and 8 interactions, respectively.

The interactions detected also depended strongly on the selection pressure: typically all screens were initially carried out without 3-aminotriazole (3AT), a competitive inhibitor of imidazoleglycerolphosphate (IGP) dehydratase, i.e. the His3 reporter enzyme used in our assays. If baits turned out to be autoactivating under these conditions, we raised the 3AT concentrations up to 50 mM in steps of 1, 3, 10, 25, and 50 mM. ORF19 was an activator at 0, 1, and 3 mM and clear results were only obtained at 10 or 25 mM (Figure 3). Most interactions disappeared at 25 mM (Figure 3B). In general, the results reported here were obtained at a 3AT concentration that clearly differentiated between signal and noise. Note that ORF19 was an activator as an N-terminal DBD-fusion but not as a C-terminal fusion (background in Figure 3A). However, this was not generally true: in 20 cases the N-terminal bait fusion autoactivated while 21 of the C-terminal baits did so. In 7 cases both fusions were autoactivators at 3 mM 3AT or higher concentrations but only one (ORF46) required more than 3 mM in both cases and was thus not interpretable (data not shown).

### Overlap between vector combinations

**Figure 4:**
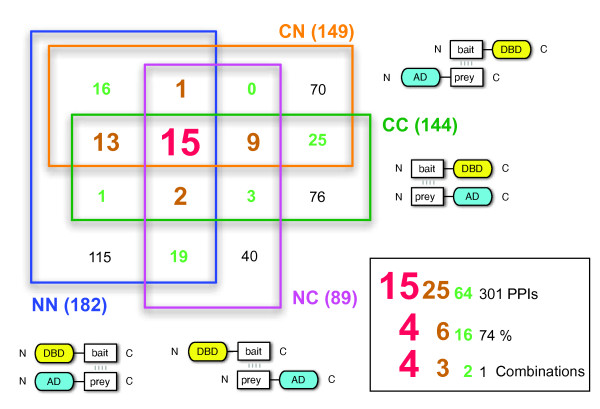
**Interactions found with combinations of N- and C-terminal fusions**. For example, 182 interactions were found with N-terminal bait and prey fusions (blue) of which 115 were only found in this combination. 15 Interactions (largest type) were found in all four combinations and are thus considered the most reliable. The box provides summaries of how many interactions (or percentages) were found in one, two, three, or four combinations.

Altogether, we have conducted more than 20,000 individual Y2H tests (96 × 96 pairwise combinations for each of the four permutations including multiple constructs for 18 proteins, see Table 1 and Additional file 1: Table S4). As shown in Figure 3 different vector combinations produced non-overlapping results. This was generally true (Figure 4): while the N-terminal fusions produced a total of 182 interactions, only 15 of them were also found with the other three combinations (Figure 4). 115 interactions were exclusively found with the N-terminal combination. No combination turned out to be superior to the other three although the number of NC pairs was somewhat lower (89) than the NN, CN, and CC pairs (ranging from 144 to 182, Figure 4). Overall, adding three additional combinations to any of the four increased the number of additional interactions by 2.2 to 4.5-fold (Additional file 1: Table S3).

**Table 1:** Overlap between screens

	NN	NC	CN	CC
NN	**182**	37 (20/42%)	45 (25/30%)	31 (17/22%)
NC	---	**89**	25 (28/17%)	29 (33/20%)
CN	---	---	**149**	62 (42/43%)
CC	---	---	---	**144**

Identical interactions found with multiple screens of different combinations. For example, the NN and NC screens found 182 and 89 interactions, respectively, of which 37 were identical (i.e. the protein pairs were identical), corresponding to 20% of NN and 42% of NC interactions.

### Reduction of false negatives

**Figure 5:**
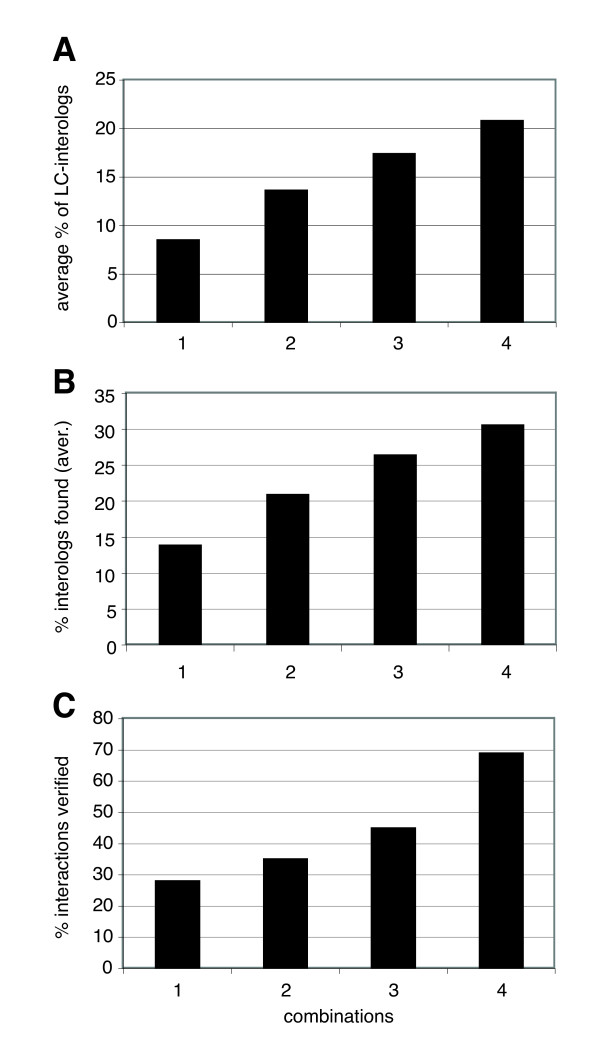
**Reducing false negatives and false positives by combining Y2H vectors**. (A) 67 interactions from five different herpesviruses curated from the literature ("LC") are compared to interactions found in this study. Shown is the fraction of these LC interactions that are found when 1, 2, 3 or 4 vector combinations are used. See text for more details. (B) Similar to (A), our VZV data was compared to large-scale Y2H interactions from HSV1, mCMV, EBV, and KSHV [4]. Shown is the fraction of interologs found by 1, 2, 3, or 4 vector combinations. See text for more details. (C) The fraction of interactions in each combination class that is supported by additional evidence, including literature-curated interactions, large-scale Y2H assays, interologs, or interactions found with alternative constructs. For example, there are 13 non-redundant interactions that have been found in all 4 combinations (NN, NC, CN, CC) of which 9 (= 69%) are supported by previously published evidence or multiple (but different) constructs. See Additional file 1: Tables S1 and S3 for details.

In order to evaluate how well our new vectors prevented false negatives we compared our data to published data. First, we used a literature-curated dataset of herpesviral-interactions [4]. Since not many VZV interactions have been published in small-scale studies that can serve as gold-standards we included small-scale interactions from other herpesviruses as well and checked whether we find interologs of these interactions (see Methods for definitions of interologs and literature-curated interactions). Fossum et al. [4] listed 91 such literature-curated interactions from five different herpesviruses including 9 in VZV. 67 of the 91 interacting pairs had both orthologs in VZV. Each of the four vector combinations found an average of 5.75 interactions out of these 67 (= 8.6%; Additional file 1: Table S3D). When data from 2, 3, or all 4 vector combinations is pooled, we detect 13.7, 17.5, and 21% of these interactions (Figure 5A). That is, the use of all four vectors more than doubled the number of known interactions detected. While 21% does not sound very impressive, one has to keep in mind that these are interologs. We do not expect that all interactions are conserved across all herpesviruses, so that much lower numbers are expected.

Similarly, we asked how many of 166 Y2H interactions found among core proteins of HSV1, mCMV, EBV, or KSHV are found in at least one of our 4 vector combinations (core proteins are those proteins that are conserved in all 5 viruses). Again, given that not all interactions are conserved, we do not expect to find all of these interactions. If single screens are performed (i.e. with only one of the 4 vector pairs) we find 14% of these core interactions on average (Figure 5B, Additional file 1: Table S3E). However, if we use all four vector combinations, this fractions more than doubles to 31% or 51 interactions. This clearly demonstrates that the use of multiple fusion proteins can significantly reduce the number of false negatives in Y2H screens.

### Reduction of false positives

It is almost impossible to prove the existence of false positives, especially for viral proteins that are known to be expressed in the same cell. We simply cannot know for sure that interactions detected in a Y2H assay do not happen in a human host cell. Thus we provide only circumstantial evidence that interactions found in multiple combinations are of higher quality than interactions found in a single screen. We looked at interactions that we found in one, two, three, or four combinations and then determined the fraction of these subsets that have been confirmed by either literature curated interologs, Y2H interologs, or independent Y2H assays. The latter include cases in which an interaction is found with different protein fragments, e.g. the interaction shown in Figure 3 involves ORF18 as well as ORF18C which is a C-terminal fragment of ORF18. We believe that interactions found with multiple protein fragments support this interaction (as long as the fragments are not exclusive as in non-overlapping N- and C-terminal fragments). Figure 5C shows that the number of combinations that an interaction was found in is directly correlated with the level of confirmation. That is, the more combinations an interaction is found with, the more likely it is to be found in our literature-curated gold standard dataset, among a set of interologs, or among additional constructs. In other words: if an interaction is found in two or more combinations it is less likely to be a false positive.

### Which vector combination is best?

We wondered whether any of the four permutations was superior to the other ones. Surprisingly, this does not seem to be the case. The fraction of validated interactions (based on interologs and validating alternative constructs) in all cases ranges from 45% to 50% (Additional file 1: Table S3C).

## Discussion

The main problem of Y2H screens is the high rate of false negatives [11,12] and new vector systems as the one shown here may alleviate that problem. We have shown recently that alternative N-terminal vector systems can reduce the number of false positives and this study extends this finding to C-terminal fusions and combinations with N-terminal vectors.

### Expression levels of bait and prey proteins

Results of Y2H assays may be affected by the expression levels of bait and prey proteins, e.g. if one or both are inefficiently translated or if the proteins themselves are unstable. We have not tested the expression levels of the various N- and C-terminal fusion proteins used here but we believe that their expression levels are similar or at least sufficient for several reasons. Most importantly, we get similar numbers of interactions with N- and C-terminal fusion proteins, indicating that they both form functional (or at least interacting) proteins, even if the NC combination yielded a significantly lower number of interations than the CN combination.

### Independent confirmation

Two-hybrid interactions have often been considered as unreliable, generating many false positive and false negative results [13]. Often false positives are suspected because many interactions are implausible and they lack independent confirmation. However, while additional assays such as co-immunoprecipitation can corroborate Y2H interactions, they are often time-consuming, expensive, and require additional reagents and protocols. In addition, a large fraction of bona fide interactions may be suitable for one particular assay but not for another and thus confirmatory experiments may still miss up to 80% of all true interactions [12]. Our results show that permutating fusion proteins in Y2H screens may mitigate these problems without requiring additional assays.

### Further permutations of the Y2H system

Obviously many more variations of the Y2H system are possible in addition to changing the location and nature of fusion tags, e.g. copy number, yeast strains, reporters etc. [1]. Other Y2H systems including commercial vectors may benefit from the permutations described and they should be used routinely in large-scale screens.

The parental vectors and the two new C-terminal vectors described here are identical in most ways, including their origins of replication (and thus their copy number), their promoters (and thus protein expression levels), as well as the yeast strains in which they were expressed. In addition, the experimental conditions of our Y2H assays were identical (except for varying 3AT concentrations). Hence, we conclude that differences in results must result from structural constraints imposed by the location of the DBD and AD fusion tags and the associated sequences.

### Novel VZV interactions: ORF10-ORF57

Interactions that are found in multiple permutations but have not been reported in the literature are the most interesting ones for follow-up studies. For space reasons we cannot discuss all interactions that we consider as well substantiated by multiple Y2H assays. One intriguing example is the interaction between ORF57 and ORF10 which we found in all four permutations. ORF10 protein is the homologue of the herpes simplex virus type 1 (HSV-1) VP16 protein. Both proteins have been shown to be structural components of the virion tegument, but also appear to activate viral immediate-early (IE) protein promoters [14]. In contrast to HSV-1, where ORF10 is essential, VZV ORF10 is dispensable for viral replication in vitro [15]. However, a more detailed analysis of VZV ORF10 deletion mutants in SCIDhu skin xenografts revealed that ORF10 is a virulence factor for the pathogenesis of VZV in skin [16,17]. VZV ORF10 mutants are characterized by decreased viral titers and decreased cutaneous lesions within skin xenografts. Electron microscopy (EM) pictures showed that VZV-infected epidermal cells had significantly fewer DNA containing nucleocapsids and extensive aggregates of intracytoplasmic viral particles [16].

Little is known about the function of the VZV ORF57 protein. This protein has been shown to localize to the cytoplasm of infected cells and to be dispensable for the replication of VZV in vitro [18]. However, UL3.5, the homologue of VZV ORF57 in pseudorabies virus (PRV), has been demonstrated to be essential for virus replication in vitro, playing an important role during viral egress. EM pictures showed that cells infected with the PRV UL3.5 deletion mutants exhibited accumulated intracytoplasmic viral capsids [19,20]. Deletion mutants of VZV ORF10 and the UL3.5 homologue of VZV ORF57 in PRV exhibit identical phenotypes, i.e. the intracytoplasmic aggregation of viral particles, and thus suggest that both proteins play an essential role in virus egress. However, their precise molecular role in this process remains to be clarified.

Our data contains a total of 15 pairs that are found in three or four permutations but have never reported in the literature (Additional file 1: **Table S1**). They should be excellent candidates for further studies of VZV molecular and structural biology.

### Recommendations for future interactome screens

As a direct result from our data we recommend that future screens should be done with a combinatin of vectors. For example, mixtures of N- and C-terminal bait plasmids could be mated with mixed N- and C-terminal prey libraries (or arrays). As with conventional screens bait constructs would have to be tested for auto-activation but this remains straight-forward. In order to improve mating efficiency the four combinations (NN, NC, CN, CC) could be mated separately and then mixed for further selection of positives. However, for less complex libraries (as those of virus genomes) this should not be necessary. Given that other factors in addition to N- or C-terminal fusions play a role in Y2H screens [1], additional vectors could be added.

## Conclusions

In summary, using permutations of fusion proteins has the potential to dramatically reduce false negatives in yeast two-hybrid screens while providing a simple way to rank interactions qualitatively. This should significantly improve future large-scale screens as well as downstream analysis of selected interactions.

## Methods

### Literature-curated interaction data and interologs

Literature-curated interactions were derived from [4]: First, a few VZV interactions from small-scale studies are considered to be highly reliable as they have been verified by additional experiments. Second, small-scale interactions from other herpesviruses with homologs in VZV are here considered as "literature-curated" as well. Third, we used large-scale Y2H data from [4] and other studies. If these interactions (in any herpesvirus different from VZV) had homologous pairs in VZV, they were considered as "interologs". For example, the KSHV interaction ORF60-ORF61 has a homologous interacting pair in VZV, ORF18-ORF19 which is thus an interolog of ORF60-ORF61.

### Vector construction and ORF cloning

Here we describe a new set of vectors that are based on the well-established pGBKT7 and pGADT7 vectors (Clontech) and their Gateway derivatives pGBKT7g and pGADT7g [21] (Figure 1). We modified the latter two vectors by inserting the Gateway RfB cassette (for reading frame B) in a way that the DBD and AD can be fused to the C-termini of cloned proteins as opposed to the usual N-termini and named these vectors pGBKCg and pGADCg, respectively (Figure 2). Next, we cloned all full-length 69 open reading frames (ORFs) as well as 18 protein fragments of Varicella-Zoster-Virus (VZV) into pGADT7g, pGBKT7g, pGADCg, and pGBKCg (Additional file 1: Table S4).

### Yeast two-hybrid assays and quality scoring

We systematically tested all N- and C-terminal baits against all N- and C-terminal VZV preys using standard matrix-based Y2H protocols as described previously [21,22]. The quality of interactions was evaluated by two different strategies: first, we compared our interactions to a list of known herpesviral interactions. Because not many interactions have been detected in VZV (except in our own studies [4,5], we used a list of herpesviral interactions curated from small-scale studies [4] as a gold-standard dataset. We considered a VZV interaction as "LC-verified" if it had at least one interolog in this literature-curated (LC) dataset. Second, we compared our VZV dataset to a large set of herpesviral Y2H interactions [4], comprising interactions from KSHV, VZV, EBV, mCMV, and HSV1. We considered a VZV interaction as "interolog-verified" if it had at least one interolog in one of the four non-VZV viruses (as VZV interactions would not be interologs). This logic is based on the idea that interactions are conserved and that multiple interologs support an interaction [23-25]. All interactions, interologs, and literature-curated interactions are available as Additional file 1: Tables S1-3 and from [4].

## Competing interests

The authors declare that they have no competing interests.

## Authors' contributions

TS carried out all cloning steps and Y2H assays. TS, RH, AB, JH, and PU analyzed data. AB, VRP, and JH cloned the VZV ORFs. PU conceived this study. PU and AB wrote the manuscript. All authors read and approved the final manuscript.

## Supplementary Material

Additional file 1 **Table S1. Y2H data - all interactions described in this paper**. **A **detailed legend is included in the Excel spreadsheet. The protein interactions from this publication have been submitted to the IMEx http://imex.sf.net consortium through IntAct (pmid: 17145710) and assigned the identifier IM-11718. The dataset is available at http://www.ebi.ac.uk/intact/search/do/search?searchString=IM-11718. Table S2. All PPIs - interactions of this study combined with all previously published interactions among herpesviral proteins. Table S3. Verifications. A. Permutations and contribution to interaction data. B. Verification by additional evidence. C. All permutations seem to be created equal. This table also contains more detailed explanations. Table S4. All VZV proteins and fragments used in this study. Click here for file
